# Potential Protective Protein Components of Cow’s Milk against Certain Tumor Entities

**DOI:** 10.3390/nu13061974

**Published:** 2021-06-08

**Authors:** Christian Leischner, Sarah Egert, Markus Burkard, Sascha Venturelli

**Affiliations:** 1 Institute of Nutritional Sciences 140, Nutritional Biochemistry 140c, University of Hohenheim, Garbenstr. 30, 70599 Stuttgart, Germany; christian.leischner@uni-hohenheim.de; 2 Institute of Nutritional Medicine, Nutritional Science/Dietetics 180c, University of Hohenheim, Fruwirthstr. 12, 70599 Stuttgart, Germany; sarah.egert@uni-hohenheim.de; 3 Department of Vegetative and Clinical Physiology, Institute of Physiology, University Hospital Tuebingen, Wilhelmstr. 56, 72074 Tuebingen, Germany

**Keywords:** cow’s milk, antitumoral effects, health-promoting diet, cancer, antitumor peptides

## Abstract

Milk and dairy products, especially from cow’s milk, play a major role in the daily human diet. It is therefore hardly surprising that the subject of milk is being extensively researched and that many effects of individual milk components have been characterized as a result. With the wealth of results available today, the influence of milk on the development of various types of cancer and, in particular, its often protective effects have been shown both in vitro and in vivo and in the evaluation of large-scale cohort and case-control studies. Various caseins, diverse whey proteins such as α-lactalbumin (α-LA), bovine α-lactalbumin made lethal to tumor cells (BAMLET), β-lactoglobulin (β-LG), or bovine serum albumin (BSA), and numerous milk fat components, such as conjugated linoleic acid (CLA), milk fat globule membrane (MFGM), or butyrate, as well as calcium and other protein components such as lactoferrin (Lf), lactoferricin (Lfcin), and casomorphines, show antitumor or cytotoxic effects on cells from different tumor entities. With regard to a balanced and health-promoting diet, milk consumption plays a major role in a global context. This work provides an overview of what is known about the antitumoral properties of proteins derived from cow’s milk and their modes of action.

## 1. Introduction

Almost all mammalian individuals including humans usually take up the preform of the mother’s milk, the so-called colostrum, which is formed by the female mammary glands after birth. A short time later, ordinary milk is produced and, in the first period, exclusively nourishes the newborn with its composition adapting to the developmental stage and growth requirements. At this time, milk serves as the sole source of nutrition until weaning containing all the ingredients required for development such as proteins, enzymes, carbohydrates, vitamins, trace elements, and, to ensure a functional immune response, antibodies as well as defense-promoting enzymes.

Humans began to build up a regulated dairy industry about 10,000 years ago with the domestication of goats and sheep, especially in Western Asia, and later, 8500 years ago, in Southern Europe with the keeping of the aurochs. In particular, the evolutionary advantage for Northern Europeans was the so-called lactase persistence, a strong positive selection of the lactase allele with no longer occurring lactose intolerance, as a result of the habituation to dairy cattle farming [1].

In recent years, global cow’s milk production has expanded from a total of 441.97 million tons in 2010 to 524.41 million tons in 2019—an increase of 18.65% over this period [2,3]. In 2019, the largest producing countries were in the EU (155.2 million tons), with the main share of 33.1 million tons in Germany [4], the USA (99.06 million tons), and India (92 million tons), with particularly strong growth rates in India (+45.3% since 2010 with 50.3 million tons) [2,3]. Per capita milk consumption including milk equivalents of dairy products without butter has increased on a global average from 78.24 kg in 2000 to 111.6 kg in 2019, which means an increase of 29.9% for this timespan [5,6]. In addition, all national and international dietary guidelines recommend a regular intake of milk and dairy products (e.g., yogurt and cheese) as part of a health-promoting eating pattern [7]. The dairy group supplies many nutrients, including calcium, phosphorus, vitamin D, vitamin A, riboflavin, vitamin B12, protein and essential amino acids, potassium, zinc, choline, magnesium, and selenium [8].

In 2020, the World Health Organization (WHO) reported a global cancer burden of 18.1 million cases (thereof 9.6 million deaths) for the year 2018 and a probable increase to 29.4 million cases in 2040. Therefore, besides its treatment, the effective primary prevention of carcinoma development is of paramount importance [9]. The highest incidence for males was found for lung, prostate, and colorectum cancers, with a proportion of 14.5%, 13.5%, and 10.9%, respectively. For females, breast, colorectum, and lung were most frequently affected (24.2%, 9.5%, and 8.4%, respectively).

For these reasons, besides the pure development of new anti-cancer drugs, emphasis has always been placed on the study of naturally occurring compounds, which often also show interesting cancer preventive effects. Some nutritional compounds succeeded, e.g., in limiting the growth of cancer cells and, at the same time, positively influenced the body’s own immune system [10,11,12,13]. In this context, daily nutrition plays an important role, which additionally increases the special interest in milk components and their effects. In addition, evidence from case-control and prospective cohort studies demonstrate an inverse association between the regular intake of milk and dairy products and the development of various types of cancer, especially of colorectal cancer [14,15,16,17]. However, causality cannot be inferred from these statistical associations. Observed inverse relations between intake of dairy products and colorectal cancer development have been largely attributed to their high calcium content. Other nutrients or bioactive constituents in dairy products, such as Lf, vitamin D or the short-chain fatty acid butyrate may also impart some protective functions against cancer, but these require much better elucidation.

## 2. Composition of Cow’s Milk

**Figure 1:**
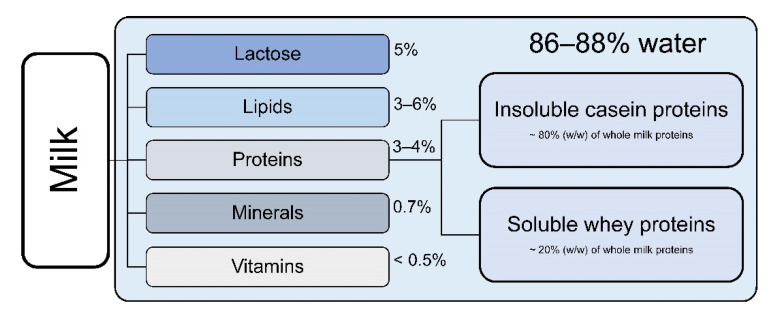
Approximate composition of cow’s milk. Proteins are divided into insoluble casein proteins and soluble whey proteins [18].

Bovine milk constituents are mainly water (86–88%), milk fat (3–6%), protein (3–4%), lactose (5%), and minerals (0.7%) as shown in Figure 1. The percentage of total solids is 11–14% [18].

**Figure 2:**
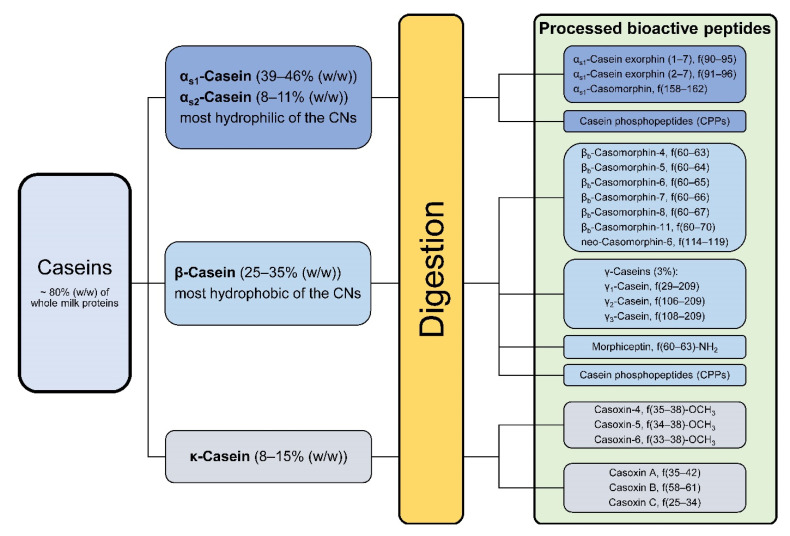
Overview of the insoluble casein proteins and their share in the total casein fraction. The green box indicates some important processed peptide variants after casein digestion. Some of them are α-amides, methyl esters or have free α-carboxyl groups [20,21,22].

Cow’s milk protein content consists of two major families of proteins. About 80% of total protein represent different insoluble caseins (CNs); the remaining 20% represent soluble whey proteins. The casein phosphoproteins can be further diversified into four families according to the homology of their primary amino acid sequences, namely α_s1_- (α_s1_-CN, 12–15 g/L in skim milk), α_s2_- (α_s2_-CN, 3–4 g/L), β- (β-CN, 9–11 g/L), and κ-caseins (κ-CN, 2–4 g/L), each consisting of several different variants due to genetic heterogeneity (Figure 2) [19].

**Figure 3:**
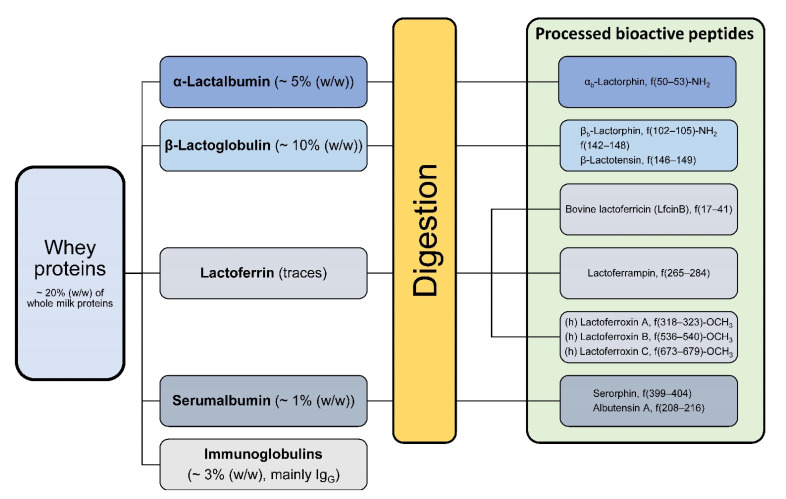
Overview of the soluble whey proteins and their share in the total protein fraction. The green box indicates some processed peptide variants after whey protein digestion. Some of them are α-amides, methyl esters or have free α-carboxyl groups [20,23].

The casein proteins occur as macromolecular aggregates known as casein micelles, with a size from 30 to 300 nm. The whey protein fraction includes α-LA (0.6–1.7 g/L), β-LG (2–4 g/L), BSA (0.4 g/L), immunoglobulins (predominantly IgG1, 0.3–0.6 g/L), bLF (0.02–0.1 g/L), lactoperoxidase, and other proteins to a minor proportion (Figure 3) [19].

## 3. Milk Proteins and Processed Peptides with Chemopreventive Properties

Milk proteins are components, which have certain physiological functionalities, that can often be diversified according to their potential to release bioactive peptides after enzymatic digestion in vitro and in vivo. Large numbers of potentially effective peptides are “encrypted” in the complete proteins until they are activated by enzymes in the gastrointestinal environment. Protein members of the casein and whey fraction with known antitumor activities are introduced in the following sections, which are additionally summarized in tabular form (Table 1).

### 3.1. Casein Proteins and Processed Peptides

#### 3.1.1. Caseins and Casomorphines

The different casein fractions occur in bovine milk in following proportions: α_s1_ (39–46% of total caseins), α_s2_ (8–11%), β (25–35%), κ (8–15%), and γ (3%), which is a natural degradation product of β-casein (Figure 2) [21,22,43,44]. Depending on cattle breed, all casein families are additionally present in multiple genetic variants with different amino acid substitutions [19], which give rise to a multitude of biologically active protein fragments after hydrolysis during gastrointestinal digestion or food processing like fermentation [20,45,46]. In milk, for example, β-caseins occur in 13 different genetic variants, of which the most common are A1 and A2 [47]. These two differ from each other only in a single amino acid substitution at position 67 (histidine in A1 β-casein to proline in A2 β-casein) caused by a single nucleotide exchange, respectively.

In vitro experiments with bovine α-, β-, and κ-casein proteins showed a reduction of migration ability of murine mammary tumor cells Met-1 and two human breast cancer cell lines (MCF10A H Ras (G12V) and MDA-MB-231) with α-casein being most potent [24]. Additionally, recombinant α-casein expression in Met-1 cells led to reduced tumor mass and lung metastasis in athymic nude mice by induced STAT1 signaling. Furthermore, different proteolytic fragments of κ- and β-casein showed antitumor effects against adenocarcinoma (MCF-7), ovarian cancer (SKOV3), or murine melanoma (B16F10) [25,26,27].

Bovine casomorphins are a group of opioid-like peptides derived from limited proteolysis of α- and β-caseins. The first casomorphin identified after an enzymatic casein digest was the heptapeptide β_b_-casomorphin-7 (BCM-7; Tyr-Pro-Phe-Pro-Gly-Pro-Ile) and the corresponding β_b_-casomorphins-4, -5, and -6 (BCM-4/-5/-6; peptides with 4, 5, and 6 amino acid residues) [48]. Opioid peptides derived from bovine α-casein- and κ-casein-digestion are named α_b_-casein exorphins (1–7 and 2–7) and casoxins A, B, and C, respectively [49]. 

In the context of antitumoral activity, different casomorphin peptides were shown to have antiproliferative effects on human prostatic cancer cell lines LNCaP, PC-3, and DU 145 through partial interaction with opioid receptors [28]. Other results showed the in vitro blocking of breast cancer T47D cells in the G0/G1 phase through antiproliferative action of five investigated casomorphins (α_s1_-caseins f(90–95) and f(90–96), BCM-7 f(60–66), BCM-5 f(60–64), and morphiceptin (amide of BCM-4)) [29]. Interaction between the casomorphins and the tumor cells takes place via δ- and κ-opioid receptors with exception of morphiceptin interacting with type II somatostatin receptor. In this case, the CMs showed different receptor affinities. In another work by Noni et al., BCM-7 is described as a weak opioid receptor agonist [50]. Furthermore, BCM-7 and the phosphopeptide β-casein f(1–25)4P have been shown to induce apoptosis of HL-60 cells (promyeloic leukemia) [30].

Nevertheless, it should be mentioned that the proliferation of prostate cancer cell lines LNCaP and PC3 was described to be increased after treatment with α-casein and total casein (0.1 or 1 mg/mL, respectively) from bovine milk. Interestingly, growth rates of lung cancer cells (A459), stomach cancer cells (SNU484), breast cancer cells (MCF7), immortalized human embryonic kidney cells (HEK293), and immortalized normal prostate cells (RWPE1) were not affected [51].

#### 3.1.2. Casein Phosphopeptides

Casein phosphopeptides (CPPs) are released by gastrointestinal trypsin digestion from α_s1_-, α_s2_-, or β-caseins (which differ in their extent of phosphorylation) and function as carriers for different minerals (like magnesium, iron, and trace elements such as zinc, barium, chrome, nickel, cobalt, and selenium) by forming complexes (especially with calcium) modulating their bioavailability [52]. Therefore, the precipitation of calcium phosphate in the intestines is inhibited and the amount of soluble calcium for absorption is increased [53]. Cytomodulatory effects of CPPs are, e.g., stimulation of interleukin-6 cytokine release in intestinal epithelial cells and stimulation of immunoglobulin A production [54,55]. CPPs are also shown to interact with voltage-operated L-type calcium channels, thereby modulating proliferation and apoptosis depending on intestinal tumor HT-29 cell differentiation [31]. CPP-induced calcium uptake is also elevated in human intestinal Caco2 cells, but only upon cell differentiation [56].

### 3.2. Whey Proteins and Processed Peptides

#### 3.2.1. Lactoferrin

Human and bovine Lf (hLf, bLf) are single chain multifunctional glycoproteins with non-haem iron-binding properties and belong to the family of transferrin proteins, which are capable of binding and transferring Fe^3+^ ions [57]. hLf and bLf consist of 689 and 691 amino acid residues, respectively, with a molecular mass of about 80 kDa [58]. Lf possesses two binding sites for one ferric ion (Fe^3+^) and one bicarbonate anion each [59]. It is produced by mucosal epithelial cells and also stored in the secondary granules of polymorphonuclear leukocytes (PMNLs) of miscellaneous mammalian species [60]. It was first purified in 1939 as unknown “red fraction” from Cow’s milk [61]. Later in 1960, it was demonstrated to be the main iron binding protein in human milk [62,63,64]. It exists as hLf and bLf with a sequence homology of nearly 70% [58]. bLf is also the main iron-binding protein in cow’s milk [65] and is present in other biological secretory fluids like saliva, tears, nasal, and bronchial secretions, as well as in semen, urine, vaginal, and gastrointestinal fluids [66,67]. Lf is fundamentally involved in the regulation of cellular and systemic iron homeostasis [68,69,70]. Caseins make up the largest share of the protein components of milk with Lf having also a large proportion, ranging from a concentration of about 7 g/L in human colostrum to 1–3 g/L in mature milk [71,72], whereas Cow’s milk contains lesser Lf, ranging from 0.05 to 0.5 g/L from early-to-mid lactation [19,73,74]. Through its antiviral, bactericidal, and antifungal properties, Lf being part of the innate immune response plays a functional role among others for the health state of the mammary gland [75,76]. Various other protective functions for Lf are described, e.g., antianemic, antioxidant, anti-inflammatory, immunomodulatory, or anticancer properties [33,40,77,78,79,80,81,82,83]. Gram-negative bacteria with negatively charged lipopolysaccharides anchored in the outer membrane, can be complexed with the highly positively charged lactoferrin, which then forms holes, through which milk lysozyme can enter and destroy the proteoglycan matrix [84]. Intact Lf is taken up in significant amounts by a specific Lf receptor (LfR) occurring in the apical membrane of the small intestine by an endocytotic process [85,86]. It must be taken into account that in infants and newborns, the intragastric pH is higher and the expression and secretion levels of the digestive enzymes are lower than in the adult individual with a matured digestive system. Therefore, non-absorbed unhydrolyzed hLf can be detected in the faecal extracts of breast-fed babies [87]. Moreover, different tissues like liver, bone, or brain and cell types like lymphocytes or fibroblasts express further LfRs [76]. Eventually, Lf is acting as a transcription factor inside the nucleus affecting immunomodulation by cytokine expression (e.g., interleukin-1β and transforming growth factor-β) or epithelial growth and differentiation [88]. Due to the high sequence homology of bLf, it was postulated to be broadly bioequivalent to hLf and was recently used in several in vivo and also clinical studies mainly to investigate its anti-inflammatory potential in oral or even aerolized form [89,90,91]. In a mouse model, orally administered bLf showed inhibitory effects on lung metastatic colony formation after subcutaneous injection of colon carcinoma Co26Lu cells [32]. Furthermore, the tumor growth and lung metastasis of murine melanoma B16 and lymphoma L5178Y-ML25 cells could be inhibited by bLf in another mouse model, interestingly mainly apo-Lf [33,92]. Moreover, Cutone and colleagues showed that native and iron-saturated bLf differently inhibited cell migration in a human glioblastoma model via the reversal of epithelial-to-mesenchymal transition-like process and inhibition of the IL-6/STAT3 pathway, with the holo-form being the more effective form [38]. These observations highlight the importance of iron saturation state of Lf when conducting in vitro and in vivo studies [68]. Additionally, Lf has been suggested to affect tumor cell growth through natural killer (NK) and lymphokine-activated killer (LAK) cell activation [92,93]. After bLf treatment, an inhibition of colon carcinoma development caused by azoxymethane administration has been observed in rats [34]. In another mouse model, elevated levels of IL-18 produced by intestinal mucosa were detectable after bLf treatment, further caspase-1 activity and interferon-γ (IFN-γ) levels were increased [94]. The proinflammatory cytokine IL-18 is an important factor in mucosal immunity by generating CD4^+^ and CD8^+^ T cells and the activation of T and NK cells followed by IFN-γ production [95,96]. Caspase-1 is necessary to process the mature active form of IL-18 and is itself activated by enzymatical cleavage of its procaspase-1 precursor [97]. Other experiments with oral bLf showed the additionally increased expression of IFN-α and IFN-β in mouse Peyer´s patches (PP) and mesenteric lymph nodes (MLN) stimulating intestine-associated immune functions [98]. hLf, with its high similarity to bLf (about 70% sequence homology and similar 3-dimensional structure) [99], was able to inhibit the cell proliferation of MDA-MB-231 breast carcinoma cells at G1 to S transition of the cell cycle by decreasing the activity of cyclin-dependent kinases [100]. Experiments with hLf demonstrated the further downregulation of 3-phosphoinositide-dependent protein kinase 1 (PDK1) transcription via mitogen-activated protein kinase/c-Jun pathway following deactivation of AKT signaling leading to inhibition of nasopharyngeal carcinoma (NPC) tumorigenesis [101]. Moreover, pepsin hydrolysates of bLf showed apoptosis induction in human myeloid leukemia cells (HL-60) and human oral squamous cell carcinoma cells SAS, demonstrating the activity of bLf inherent peptides [35,36]. In an in vivo experimental setting with hamsters, the incidence of 7,12 dimethylbenz[a]anthracene (DMBA)-induced hamster buccal pouch (HBP) carcinogenesis could be decreased by feeding a basal diet containing 0.2% bLf. In this case, levels of phase I enzymes were decreased, lipid peroxidation was modulated, and antioxidant and phase II enzyme activities increased [37]. In a rat hepatocellular cancer model, Lf showed chemopreventive effects by regulating protein kinase B pathway [102] and liposomal bLf was described to suppress inflammation and tumor cell proliferation in a 1,2-dimethylhydrazine/dextran sulphate sodium (DMH-DSS)-induced colorectal cancer rat model [39]. Notably, no adverse effects were described after applying 1.5–9 g recombinant hLf in a phase I trial to treat refractory solid tumors [103]. 

#### 3.2.2. Lactoferricin

In adulthood, bLf ingested orally by drinking Cow’s milk is largely enzymatically cleaved into smaller peptides by gastric pepsin in the stomach with a low pH of 1.0–2.5 [104], followed by losing some of its abilities like binding iron or a comparably lower anti-viral activity. Nonetheless, degradation products such as bovine lactoferricin (LfcinB, 25 amino acid residues 17–41 of bLf) or lactoferrampin (20 amino acid residues 265–284 of bLf) are amphipathic peptides with the more hydrophobic residues lying on one side, more positively charged residues on the other [105], and possess antimicrobial activity. Lactoferrampin differs by showing more antimicrobial properties, whereas Lfcin additionally exhibits anti-inflammatory and anticancer activities [105]. The antitumor effects of LfcinB are described against a variety of tumor entities, like murine leukemia, fibrosarcoma (Meth A), melanoma (B16F10), or coloncarcinoma (C26) [40]. Lfcin disrupts the cell membrane and triggers apoptosis through an oxidant-dependent pathway. The amphipathic structure is necessary to enter the cell by targeting the peptide to the relatively negative charged cell surface because of more abundantly present phosphatidylserine molecules [106] followed by the insertion of the hydrophobic residues into the membrane disrupting cell integrity by pore formation [40].

#### 3.2.3. α-Lactalbumin and Bovine α-Lactalbumin Made Lethal to Tumor Cells (BAMLET)

α-LA is the second most common whey protein following β-LG in human and bovine milk and has a molecular weight of 14.2 kDa (123 amino acid residues). It is produced in the lactating mammary gland of almost all mammals and is required for the synthesis of lactose [107]. Human and bovine α-LA share 71% sequence homology and similar Ca^2+^-binding sites [108].

Back in 1995, the human equivalent of BAMLET called human α-lactalbumin made lethal to tumor cells (HAMLET) was discovered to kill a variety of transformed, embryonic, and lymphoid cells sparing mature epithelial cells [109]. Later, it was shown that the fatty acid oleic acid (C18:1) was necessary as cofactor to form an effective complex with α-LA to induce apoptosis [110]. Hereby, the folding and stability of α-LA is affected by pH and Ca^2+^ ions and play a crucial role in forming BAMLET. α-LA must be present in the partially unfolded apo-state (devoid of Ca^2+^) before complex formation [110]. The observed cytotoxicity against tumor cells by inducing cell death works via lysosomal membrane permeabilization and was demonstrated by experiments from Rammer and colleagues. According to their results, this is due to the specific accumulation of BAMLET in the endolysosomal compartment of tumor cells, followed by the release of cathepsins and other lysosomal hydrolases into the cytosol, which activates proapoptotic Bax protein [41]. Thereby, cytotoxicity against a variety of human (HeLa, J82, RT4, PC-3, U118, MCF-7, and U2-OS) and murine (L1210) tumor cells was demonstrated, likewise low cytotoxicity against healthy murine embryonic fibroblasts (NIH-3T3). However, the results of various groups that also show the cytotoxicity of BAMLET to primary cells should not be ignored. Brinkmann et al. showed that peripheral blood mononuclear cells (LC_50_ of 20 µg/mL BAMLET) were quite sensitive against BAMLET treatment compared with primary endothelial cells (LC_50_ of 1.37 mg/mL), in addition to be cytotoxic against different carcinoma cell lines (e.g., HL-60 (human promyeloic leukemia), Skov-3 (human ovarian adenocarcinoma), and B16F0 (murine melanoma) [111]. In these experiments, α-LA alone was shown to be not toxic, otherwise oleic acid treatment of Jurkat (acute T cell leukemia) and THP1 cells (acute monocytic leukemia) alone led to cell death resembling apoptosis and necrosis.

#### 3.2.4. β-Lactoglobulin

β-LG is the most abundant non-casein protein in bovine milk, with a share of about 50% of the whey protein fraction, and is not present in human milk. Its molecular weight is 18.3 kDa (162 amino acid residues) and it exists as a dimer of two main genetic variants A and B, which differ by two-point mutations under physiological pH and ambient temperature [23]. Below pH 3, β-LG dissociates into monomers [112]. The protein is thought to act as whey carrier protein for a variety of hydrophobic molecules, like retinoids (e.g., vitamin A), lipids, and polyphenols protecting them against oxidative damage or increasing their solubility [113]. For ligand binding, pH is important influencing the access to and release from the hydrophobic binding pocket [114]. β-LG is quite resistant to peptic and chymotryptic digestion, because of its structural and conformational properties where the cleavage sites are not easily accessible [115]. Therefore, β-LG could eventually survive the gastrointestinal passage unharmed and increase intestinal absorption of protected bound molecules. Anti-tumor effects have been seen in vitro within a study with different human tumor models (lung tumor cell line A549, intestinal epithelial tumor cell line HT-29, hepatocellular cell line HepG2, and breast cancer cell line MDA231-LM2) and further in vivo in a xenograft model with BALB/c nude mice with orally administered protein [42]. Thereby, tumor growth and development were reduced through induction of mitochondria-dependent apoptosis by upregulation of Bax and Caspase-3 levels and decreasing Bcl-2 level. Furthermore, β-LG was used as nanocarrier for hydrophobic acid labile drugs for oral administration like irinotecan, a potent agent in colorectal cancer treatment, which showed more cytotoxic effectivity against human gastric carcinoma AGS cells and colon carcinoma HT-29 cells than the free drug [116]. Interestingly, β-LG was shown to form cytotoxic complexes by binding sodium oleate analogously to the HAMLET/BAMLET complexes of α-LA with oleic acid [117]. Results with human monocytic cells U937 (myeloid leukemia) suggested an apoptotic pathway, whereas healthy cells were less affected, as demonstrated with rat adrenal pheochromocytoma cells PC12, which exhibit phenotypic features of mature normal cells in the differentiated state.

### 3.3. Milk Fat Globule Membrane

Milk fat globules have a unique colloidal assembly structure to pack and release triacylglycerols and other bioactive molecules in the form of droplets in milk. The functional milk fat-encapsulating membrane called milk fat globule membrane (MFGM) consists of a phospholipid trilayer. The lipid core is covered with a monolayer surface coat of proteins and polar lipids after being released from the endoplasmic reticulum into the cytosol. These microlipid droplets (MLDs) fuse together intracellularly to form larger cytoplasmatic lipid droplets (CLDs, average 3–4 µm in diameter) and are probably gradually coated with plasma bilayer membrane during release into the alveolar lumen of the mammary gland [118]. This ensures the dispensation of the droplets in milk serum without aggregation with others. Proteome analysis of bovine MFGM revealed a composition of 69–73% lipid and 22–24% protein [119]. Major proteins are, e.g., periodic acid Schiff 6/7 (PAS6/7, the bovine homologue of human lactadherin), butyrophilin (BTN), or mucin-1 (MUC1) and play a role in the fight against bacteria and viruses [120]. Among the multiple proteins identified, almost half of them exert membrane/protein trafficking or cell signaling functions [121]. In vitro experiments with MFGM isolates in a colon cancer cell model with human HT-29 cells revealed varying antiproliferative capacities of differently processed samples. Both thermal denaturation and hydrolysis using trypsin or phospholipase A2, affecting the protein and phospholipid fraction, respectively, led to a reduction of MFGM-mediated antiproliferative activity, suggesting the responsibility of not only the components, but also the structure of MFGM [122]. One possible mechanism among others could be the degradation of xanthine oxidoreductase (XO), a superoxide- (O_2_^−^) and H_2_O_2_-producing molybdenum flavoprotein with low specificity, which is also one of the main components of the MFGM [123].

## 4. Conclusions

The steadily increasing knowledge about bioactive peptides that originate from different food sources is a research topic that can provide lead structures or identify compounds that are suitable for the prevention or even treatment of numerous diseases. In recent years, milk has gained growing interest due to its potential health-promoting effects, which is illustrated by elevated numbers of publications highlighting anticancer activities of its compounds. In addition, there is evidence from observational studies demonstrating an inverse association between the regular intake of dairy products and colorectal cancer. Together with other well-known milk ingredients (e.g., milk lipids), the role of caseins, whey proteins, and their derivatives in the field of regulation of immunity, prevention of infection, antioxidant and anti-inflammatory effects, and anticarcinogenic action is becoming more and more evident.

Nonetheless, there are also studies that show an increase in cancer risk from milk. For prostate and breast cancer in particular, there are in vitro data as well as results from cohort studies that show the inductive effects of milk on the development of cancer [124,125]. Especially in the case of prostate cancer induction, data diverge widely between increased consumption of reduced-fat milk and whole milk [126,127]. In vitro digested Cow’s milk was also shown to particularly stimulate growth of prostate cancer cells among other investigated cells of different tumor entities in culture [128]. Further experiments concerning the effects of whole milk on the prostate carcinogenesis in rats revealed an increased tumor incidence [129]. Some studies indicate an inductive effect of milk consumption on the risk of developing breast cancer, although in turn the data situation is less clear in other studies [130,131,132]. In the case of individuals with lactose intolerance characterized by low consumption of milk and other dairy products, a decreased risk of developing lung, breast, and ovarian cancer was determined [133]. In this context, an increased incidence may be possibly suspected in hormone-associated tumors.

On the other side, many of the milk ingredients discussed in this work show promising anticancer properties, and hence, these results could play an important role in the development of chemopreventive peptide-based drugs, which are not genotoxic, have high selectivity, and are well tolerated. Nevertheless, it should always be considered whether orally administered milk proteins “survive” the gastrointestinal digestion pathway as a whole or are hydrolyzed to form smaller peptides with changed properties and special degradation patterns. This depends on their three-dimensional structure with differently accessible cleavage sites for the digestive enzymes, the gastric emptying time, or intragastric pH, which is different in the fasting state (in adults pH 5.0–6.0) and takes up to 100 min to reach the pH optimum for pepsin digestion of 1.5–2.0 [134]. The digestion rate therefore also depends on the individuals age, the fasting/feeding state, the enzyme:substrate ratio, and the form of administration. Hence, liquid food has a faster gastric emptying time compared to solid products. Furthermore, milk is subjected to industrial processing steps like homogenization or heating and cooling during pasteurization and ultra-high temperature treatment for sterilization, which influences protein digestibility in advance prior to consumption. To ensure gastrointestinal passage for sensitive proteins methods like microencapsulation, the covalent attachment of polyethylene glycol (PEG), named PEGylation, or saturation of ion binding sites to reduce or prevent proteolysis can be used. 

There are also differences between organic and conventionally produced milk, mainly depending on feeding patterns (e.g., year-round pasture grazing) [135]. However, the majority of studies focused on fatty acid distribution with increased levels of beneficial polyunsaturated fatty acids, such as conjugated linoleic acid in organic milk [136], with a pasture-based diet identified as a main contributor to these effects [137]. On the other hand, pasture feeding is not exclusive for organic produced milk and the differences in milk composition are diminished if this type of feeding is also used in the case of conventionally produced milk [135]. A study that compared both organic and conventional farming with year-round pasture grazing showed that other factors, such as the clover content of the pasture, can have a greater influence on milk composition than farming systems themselves [135]. Another study evaluated differences of protein content between cow’s milk from late pasture and early indoor feeding season. The authors found significant differences between organic and conventionally manufactured milk in the late pasture season regarding the beneficial proteins Lf (334.99 and 188.02 mg/L), β-LG (4.12 and 2.68 g/L), and lysozyme (15.68 and 12.56 µg/L), whereas the situation was largely different in early indoor feeding season with lower concentrations of BSA, bLf, and lysozyme in organically manufactured milk [138]. These findings highlight the complexity of the comparative effects on milk composition between organic and conventional farming, due to multiple parameters such as feed composition, seasonal effects, cow breed, or the possibility of free-range farming. In addition to identifying the possible health-promoting and anticarcinogenic properties of individual milk proteins, their quantification in milk of different species is also gaining importance. For example, goat milk contains far more Lf than cow milk, while the Lf content of human and sheep milk is also on a high level [139]. 

A similar trend can be seen in the evaluation of the milk protein content of different dairy products, such as yogurt, cheese, and further fermented dairy products. Among these, cheese, e.g., is characterized by a particularly high antioxidative potential, which is derived, among other things, from the protein fraction of the milk, and in particular, the caseins, but is also co-determined by conjugated linoleic acid, coenzyme Q, and various vitamins. Moreover, fermentation by proteolytic cleavage by probiotics seems to increase the release of antioxidant peptide fragments [140]. For instance, Lfcin is hydrolyzed by lactic acid bacteria [141]. In some studies, bLf contents of liquid milk and yogurt were found to be at a comparable level and enriched in whole milk powder [142], whereas Lf content of certain yogurts was relatively low in another study [143]. This also opens up important new research areas, for example identifying yogurt cultures that metabolize the desired milk proteins only to a small extent. Similarly, the opportunity of enriching certain components in certain modified dairy products is also an interesting approach for the production of functional food. 

According to the multiple possible interactions on different levels of cell biology and to determine exactly which milk ingredients have which influence on cancer development, there is still a lot of research that has to be done on this promising research area—on the molecular level in vitro as well as within studies of the whole organism in vivo.

## Figures and Tables

**Table 1:** Effects and potential mechanisms of milk proteins or their processed peptides with known antitumor activities against different tumor species shown by in vitro and in vivo experiments. Species are marked in brackets, respectively.

Proteins			Tumor Species	Effects and Potential Mechanism	Ref.
Casein proteins	CNs	α-, β-, κ-caseins	Breast cancer (human): MCF10A H Ras (G12V), MDA-MB-231	Decreased cell migration	[24]
			Mammary tumor (murine): Met-1	Decreased cell migration, tumor growth, Metastasis, activation of STAT1 signaling, apoptosis induction (shown for α-CN)	
		Lactaptin (κ-casein fragment)	Breast carcinoma (human): MCF-7	Apoptosis induction	[25]
		PGPIPN (β-casein fragment)	Ovarian cancer (human): SKOV3	BCL2 targeting	[26]
		INKKI (β-casein fragment)	Melanoma (murine): B16F10	Apoptosis induction	[27]
	CMs		Prostatic cancer (human): LNCaP, PC-3, DU145	Interaction with opioid receptors	[28]
			Breast cancer (human): T47D	G0/G1 blocking	[29]
			Promyeloic leukemia (human): HL-60	Apoptosis induction	[30]
	CPPs		Intestinal tumor (human): HT-29	Interaction with voltage-operated L-type calcium channels, apoptosis	[31]
Whey proteins	Lf		Colon carcinoma (murine): Co26Lu	Inhibitory effects on lung metastatic colony formation in Balb/c mice due to Tand NK cell activation	[32]
			Melanoma (murine): B16-BL6	Inhibition of lung metastasis in C57BL/6 mice (only apo-Lf)	[33]
			Lymphoma (murine): L5178Y-ML25	Inhibition of liver and spleen metastasis in C57BL/6 mice (only apo-Lf)	
			Colon carcinoma (murine)	Reduced induction of aberrant crypt foci (ACF) by azoxymethane administration in male F344 rats	[34]
		Pepsin hydrolysate f(17–38)	Promyeloic leukemia (human): HL-60	Apoptosis induction	[35]
		Pepsin hydrolysate (mixture)	Oral squamous cell carcinoma (human): SAS	Apoptosis induction by JNK/SAPK MAP kinase activation	[36]
		Basal diet with 0.2% bLf	Hamster buccal pouch (HBP) carcinoma	Decreased incidence of DMBA-induced carcinogenesis, decreased levels of phase I enzymes, modulated lipid peroxidation, increased antioxidant and phase II enzyme activities	[37]
		Native and iron saturated bLf	Glioblastoma (human): GL-15	Down-regulation of Snail and vimentin expression, increase in cadherin levels Inhibition of EMT-like processes and IL-6/STAT3 axis mainly by the holo-form	[38]
		Liposomal bLf	Colorectal cancer (rat): DMF-DSS induced colorectal	Suppression of inflammation and tumor cell proliferation	[39]
	Lfcin		Fibrosarcoma (murine): Meth A	Reduction of tumor growth in CB6 mice Cytotoxic activity, lysis by pore formation (SEM)	[40]
			Melanoma (murine): B16F10	Cytotoxic activity	
			Colon carcinoma (murine): C26	Cytotoxic activity	
	α-LA in complex with oleic acid (BAMLET)		Cervical epithelial carcinoma (human): HeLa	Accumulation in endolysosomal compartment, lysosomal membrane permeabilization inducing nonapoptotic lysosomal cell death	[41]
			Bladder carcinoma (human): J82, RT4		
			Prostate carcinoma (human): PC-3		
			Astrocytoma (human): U118		
			Breast carcinoma (human): MCF-7		
			Osteosarcoma (human): U2-OS		
			Lymphocytic leukemia (murine): L1210		
	β-LG		Lung adenocarcinoma (human): A549	Apoptosis induction, upregulation of Bax and caspase-3, decreased level of Bcl-2, reduced chemotactic motility, tumor inhibition in BALB7c mice after oral administration	[42]
			Intestinal tumor (human): HT-29		
			Hepatoblastoma (human): HepG2		
			Breast carcinoma (human): MDA231-LM2		

BAMLET: bovine α-lactalbumin made lethal to tumor cells; CM: casomorphin; CN: casein; CPP: casein phosphopeptide; DMBA, 7,12dimethylbenz[a]anthracene; DMF-DSS: 1,2-dimethylhydrazine/dextran sulphate sodium, EMT: epithelial-to-mesenchymal transition, IL-6: interleukin-6, JNK/SAPK, c-Jun N-terminal kinase/stress-activated protein kinase; Lf: lactoferrin; Lfcin: lactoferricin; SEM, scanning electron microscope; STAT1/3: signal transducer and activator of transcription 1/3; α-LA: α-lactalbumin; β-LG: β-lactoglobulin.

## Abbreviations

BAMLET	bovine α-lactalbumin made lethal to tumor cells
BCM	β_b_-casomorphin
bLf	bovine lactoferrin
BSA	bovine serum albumin
BTN	butyrophilin
CLA	conjugated linoleic acid
CLD	cytoplasmatic lipid droplet
CN	casein
CPP	caseinphosphopeptide
DMBA	7,12 dimethylbenz[a]anthracene
DMH-DSS	1,2-dimethylhydrazine/dextran sulphate sodium
FAO	Food and Agriculture Organization of the United Nations
FAS	Foreign Agricultural Service
HAMLET	human α-lactalbumin made lethal to tumor cells
HBP	hamster buccal pouch
hLf	human lactoferrin
LAK	lymphokine-activated killer
Lf	lactoferrin
Lfcin	lactoferricin
LfcinB	bovine lactoferricin
LfR	Lf receptor
MFGM	milk fat globule membrane
MLD	microlipid droplet
MLN	mesenteric lymph nodes
MUC1	mucin-1
NK	natural killer
PAS6/7	periodic acid Schiff 6/7
NPC	nasopharyngeal carcinoma
PDK1	3-phosphoinositide-dependent protein kinase 1
PEG	polyethylene glycol
PMNL	polymorphonuclear leukocyte
PP	Peyer´s patches
USDA	United States Department of Agriculture
WHO	World Health Organization
XO	xanthine oxidoreductase
α-LA	α-lactalbumin
β-LG	β-lactoglobulin

## References

[1] Itan Y., Powell A., Beaumont M.A., Burger J., Thomas M.G. (2009). The Origins of Lactase Persistence in Europe. PLoS Comput. Biol..

[2] United States Department of Agriculture (USDA), Foreign Agricultural Service (FAS) Dairy: World Markets and Trade. Release Date: December 2014. https://downloads.usda.library.cornell.edu/usda-esmis/files/5t34sj56t/f4752h17r/05741s112/dairy-market-12-16-2014.pdf.

[3] United States Department of Agriculture (USDA), Foreign Agricultural Service (FAS) Dairy: World Markets and Trade. Release Date: December 2020. https://apps.fas.usda.gov/psdonline/circulars/dairy.pdf.

[4] Bundesanstalt für Landwirtschaft und Ernährung (BLE), Bundesinformationszentrum Landwirtschaft Bericht zur Markt- und Versorgungslage mit Milch und Milcherzeugnissen. Release Date: 8 May 2020. https://www.ble.de/SharedDocs/Downloads/DE/BZL/Daten-Berichte/MilchUndMilcherzeugnisse/JaehrlicheErgebnisse/Deutschland/2020BerichtMilch.pdf?__blob=publicationFile&v=2.

[5] Food and Agriculture Organization of the United Nation Agricultural Data. http://www.fao.org/faostat/en/#data/QC.

[6] Food and Agriculture Organization of the United Nations (2020). Food Outlook. Biannual Report on Global Food Markets.

[7] Oberritter H., Schäbethal K., von Ruesten A., Boeing H. (2013). The DGE Nutrition Circle—Presentation and Basis of the Food-Related Recommendations from the German Nutrition Society (DGE). Ernaehrungs Umsch. Int..

[8] Committee D.G.A. (2016). Dietary Guidelines for Americans 2015–2020.

[9] World Health Organization (2020). WHO Report on Cancer: Setting Priorities, Investing Wisely and Providing Care for All.

[10] Burkard M., Leischner C., Lauer U.M., Busch C., Venturelli S., Frank J. (2017). Dietary flavonoids and modulation of natural killer cells: Implications in malignant and viral diseases. J. Nutr. Biochem..

[11] Venturelli S., Burkard M., Biendl M., Lauer U.M., Frank J., Busch C. (2016). Prenylated chalcones and flavonoids for the prevention and treatment of cancer. Nutrition.

[12] Leischner C., Burkard M., Pfeiffer M.M., Lauer U.M., Busch C., Venturelli S. (2015). Nutritional immunology: Function of natural killer cells and their modulation by resveratrol for cancer prevention and treatment. Nutr. J..

[13] Busch C., Burkard M., Leischner C., Lauer U.M., Frank J., Venturelli S. (2015). Epigenetic activities of flavonoids in the prevention and treatment of cancer. Clin. Epigenetics.

[14] Barrubés L., Babio N., Becerra-Tomás N., Rosique-Esteban N., Salas-Salvadó J. (2019). Association Between Dairy Product Consumption and Colorectal Cancer Risk in Adults: A Systematic Review and Meta-Analysis of Epidemiologic Studies. Adv. Nutr..

[15] Savaiano D.A., Hutkins R.W. (2021). Yogurt, cultured fermented milk, and health: A systematic review. Nutr. Rev..

[16] Bermejo L.M., López-Plaza B., Santurino C., Cavero-Redondo I., Gómez-Candela C. (2019). Milk and Dairy Product Consumption and Bladder Cancer Risk: A Systematic Review and Meta-Analysis of Observational Studies. Adv. Nutr..

[17] Schwingshackl L., Schwedhelm C., Hoffmann G., Knüppel S., Preterre A.L., Iqbal K., Bechthold A., De Henauw S., Michels N., Devleesschauwer B. (2018). Food groups and risk of colorectal cancer. Int. J. Cancer.

[18] Eskin N.A.M., Goff H.D., Eskin N.M., Shahidi F. (2013). Chapter 4—Milk. Biochemistry of Foods.

[19] Farrell H., Jimenez-Flores R., Bleck G., Brown E., Butler J., Creamer L., Hicks C., Hollar C., Ng-Kwai-Hang K., Swaisgood H. (2004). Nomenclature of the Proteins of Cows’ Milk—Sixth Revision. J. Dairy Sci..

[20] Park Y.W., Nam M.S. (2015). Bioactive Peptides in Milk and Dairy Products: A Review. Food Sci. Anim. Resour..

[21] Eigel W., Butler J., Ernstrom C., Farrell H., Harwalkar V., Jenness R., Whitney R.M. (1984). Nomenclature of Proteins of Cow’s Milk: Fifth Revision. J. Dairy Sci..

[22] Fuquay J.W., Roginski H., Fox P.F. (2003). Encyclopedia of Dairy Sciences.

[23] Fiocchi A., Brozek J., Schünemann H., Bahna S.L., von Berg A., Beyer K., Bozzola M., Bradsher J., Compalati E., Ebisawa M. (2010). World Allergy Organization (WAO) Diagnosis and Rationale for Action against Cow’s Milk Allergy (DRACMA) Guidelines. World Allergy Organ. J..

[24] Bonuccelli G., Castello-Cros R., Capozza F., Martinez-Outschoorn U.E., Lin Z., Tsirigos A., Xuanmao J., Whitaker-Menezes D., Howell A., Lisanti M.P. (2012). The milk protein α-casein functions as a tumor suppressor via activation of STAT1 signaling, effectively preventing breast cancer tumor growth and metastasis. Cell Cycle.

[25] Nekipelaya V.V., Semenov D.V., Potapenko M.O., Kuligina E.V., Kit Y.U.Y., Romanova I.V., Richter V.A. (2008). Lactaptin is a human milk protein inducing apoptosis of MCF-7 adenocarcinoma cells. Dokl. Biochem. Biophys..

[26] Wang W., Gu F., Wei C., Tang Y., Zheng X., Ren M., Qin Y. (2013). PGPIPN, a Therapeutic Hexapeptide, Suppressed Human Ovarian Cancer Growth by Targeting BCL2. PLoS ONE.

[27] Azevedo R.A., Ferreira A.K., Auada A.V.V., Pasqualoto K.F.M., Marques-Porto R., Maria D.A., Lebrun I. (2012). Antitumor Effect of Cationic INKKI Peptide from Bovine β-Casein on Melanoma B16F10. J. Cancer Ther..

[28] Kampa M., Bakogeorgou E., Hatzoglou A., Damianaki A., Martin P.-M., Castanas E. (1997). Opioid alkaloids and casomorphin peptides decrease the proliferation of prostatic cancer cell lines (LNCaP, PC3 and DU145) through a partial interaction with opioid receptors. Eur. J. Pharmacol..

[29] Hatzoglou A., Bakogeorgou E., Hatzoglou C., Martin P.-M., Castanas E. (1996). Antiproliferative and receptor binding properties of α- and β-casomorphins in the T47D human breast cancer cell line. Eur. J. Pharmacol..

[30] Hata I., Higashiyama S., Otani H. (1998). Identification of a phosphopeptide in bovine αs1-casein digest as a factor influencing proliferation and immunoglobulin production in lymphocyte cultures. J. Dairy Res..

[31] Perego S., Cosentino S., Fiorilli A., Tettamanti G., Ferraretto A. (2012). Casein phosphopeptides modulate proliferation and apoptosis in HT-29 cell line through their interaction with voltage-operated L-type calcium channels. J. Nutr. Biochem..

[32] Iigo M., Kuhara T., Ushida Y., Sekine K., Moore M.A., Tsuda H. (1999). Inhibitory effects of bovine lactoferrin on colon carcinoma 26 lung metastasis in mice. Clin. Exp. Metastasis.

[33] Yoo Y.-C., Watanabe S., Watanabe R., Hata K., Shimazaki K.-I., Azuma I. (1997). Bovine Lactoferrin and Lactoferricin, a Peptide Derived from Bovine Lactoferrin, Inhibit Tumor Metastasis in Mice. Jpn. J. Cancer Res..

[34] Sekine K., Ushida Y., Kuhara T., Iigo M., Baba-Toriyama H., Moore M., Murakoshi M., Satomi Y., Nishino H., Kakizoe T. (1997). Inhibition of initiation and early stage development of aberrant crypt foci and enhanced natural killer activity in male rats administered bovine lactoferrin concomitantly with azoxymethane. Cancer Lett..

[35] Roy M., Kuwabara Y., Hara K., Watanabe Y., Tamai Y. (2002). Peptides From the N-terminal End of Bovine Lactoferrin Induce Apoptosis in Human Leukemic (HL-60) Cells. J. Dairy Sci..

[36] Sakai T., Banno Y., Kato Y., Nozawa Y., Kawaguchi M. (2005). Pepsin-Digested Bovine Lactoferrin Induces Apoptotic Cell Death With JNK/SAPK Activation in Oral Cancer Cells. J. Pharmacol. Sci..

[37] Mohan K.V.P.C., Kumaraguruparan R., Prathiba D., Nagini S. (2006). Modulation of xenobiotic-metabolizing enzymes and redox status during chemoprevention of hamster buccal carcinogenesis by bovine lactoferrin. Nutrition.

[38] Cutone A., Colella B., Pagliaro A., Rosa L., Lepanto M.S., di Patti M.C.B., Valenti P., Di Bartolomeo S., Musci G. (2020). Native and iron-saturated bovine lactoferrin differently hinder migration in a model of human glioblastoma by reverting epithelial-to-mesenchymal transition-like process and inhibiting interleukin-6/STAT3 axis. Cell. Signal..

[39] Sugihara Y., Zuo X., Takata T., Jin S., Miyauti M., Isikado A., Imanaka H., Tatsuka M., Qi G., Shimamoto F. (2017). Inhibition of DMH-DSS-induced colorectal cancer by liposomal bovine lactoferrin in rats. Oncol. Lett..

[40] Eliassen L.T., Berge G., Sveinbjørnsson B., Svendsen J.S., Vorland L.H., Rekdal Ø. (2002). Evidence for a direct antitumor mecha-nism of action of bovine lactoferricin. Anticancer. Res..

[41] Rammer P., Groth-Pedersen L., Kirkegaard T., Daugaard M., Rytter A., Szyniarowski P., Høyer-Hansen M., Povlsen L.K., Nylandsted J., Larsen J.E. (2010). BAMLET Activates a Lysosomal Cell Death Program in Cancer Cells. Mol. Cancer Ther..

[42] Li H., Li P., Yang H., Wang Y., Huang G., Wang J., Zheng N. (2019). Investigation and comparison of the anti-tumor activities of lactoferrin, α-lactalbumin, and β-lactoglobulin in A549, HT29, HepG2, and MDA231-LM2 tumor models. J. Dairy Sci..

[43] Davies D.T., Law A.J.R. (1980). The content and composition of protein in creamery milks in south-west Scotland. J. Dairy Res..

[44] Miller M.J.S., Witherly S.A., Clark D.A. (1990). Casein: A Milk Protein with Diverse Biologic Consequences. Exp. Biol. Med..

[45] Korhonen H., Pihlanto-Leppäla A., Rantamäki P., Tupasela T. (1998). Impact of processing on bioactive proteins and peptides. Trends Food Sci. Technol..

[46] Gobbetti M., Stepaniak L., De Angelis M., Corsetti A., Di Cagno R. (2002). Latent Bioactive Peptides in Milk Proteins: Proteolytic Activation and Significance in Dairy Processing. Crit. Rev. Food Sci. Nutr..

[47] Kamiński S., Cieślińska A., Kostyra E. (2007). Polymorphism of bovine beta-casein and its potential effect on human health. J. Appl. Genet..

[48] Henschen A., Lottspeich F., Brantl V., Teschemacher H. (1979). Novel opioid peptides derived from casein (beta-casomorphins). II. Structure of active components from bovine casein peptone. Hoppe-Seyler´s Zeitschrift für physiologische Chemie.

[49] Liu Z., Udenigwe C.C. (2018). Role of food-derived opioid peptides in the central nervous and gastrointestinal systems. J. Food Biochem..

[50] Noni I., FitzGerald R., Korhonen H., Livesey C., Thorsdottir I., Tomé D., Witkamp R. (2009). Review of the potential health impact of β-casomorphins and related peptides. EFSA Sci. Rep..

[51] Park S.-W., Kim J.-Y., Kim Y.-S., Lee S.J., Chung M.K., Lee S.D. (2014). A Milk Protein, Casein, as a Proliferation Promoting Factor in Prostate Cancer Cells. World J. Men Health.

[52] Meisel H., Frister H. (1988). Chemical Characterization of a Caseinophosphopeptide Isolated from in vivo Digests of a Casein Diet. Biol. Chem. Hoppe-Seyler.

[53] Berrocal R., Chanton S., Juillerat M.A., Favillare B., Scherz J.-C., Jost R. (1989). Tryptic phosphopeptides from whole casein. II. Physicochemical properties related to the solubilization of calcium. J. Dairy Res..

[54] Kitts D.D., Nakamura S. (2005). Calcium-enriched casein phosphopeptide stimulates release of IL-6 cytokine in human epithelial intestinal cell line. J. Dairy Res..

[55] Otani H., Watanabe T., Tashiro Y. (2001). Effects of Bovine β-Casein (1-28) and Its Chemically Synthesized Partial Fragments on Proliferative Responses and Immunoglobulin Production in Mouse Spleen Cell Cultures. Biosci. Biotechnol. Biochem..

[56] Cosentino S., Gravaghi C., Donetti E., Donida B.M., Lombardi G., Bedoni M., Fiorilli A., Tettamanti G., Ferraretto A. (2010). Caseinphosphopeptide-induced calcium uptake in human intestinal cell lines HT-29 and Caco2 is correlated to cellular differentiation. J. Nutr. Biochem..

[57] Metz-Boutigue M.-H., Jolles J., Mazurier J., Schoentgen F., Legrand D., Spik G., Montreuil J., Jolles P. (1984). Human lactotransferrin: Amino acid sequence and structural comparisons with other transferrins. JBIC J. Biol. Inorg. Chem..

[58] Pierce A., Colavizza D., Benaissa M., Maes P., Tartar A., Montreuil J., Spik G. (1991). Molecular cloning and sequence analysis of bovine lactotransferrin. JBIC J. Biol. Inorg. Chem..

[59] Anderson B.F., Baker H.M., Norris G.E., Rice D.W., Baker E.N. (1989). Structure of human lactoferrin: Crystallographic structure analysis and refinement at 2·8 Å resolution. J. Mol. Biol..

[60] Masson P.L., Heremans J.F., Schonne E. (1969). Lactoferrin, an iron-binbing protein in neutrophilic leukocytes. J. Exp. Med..

[61] Sørensen M., Sørensen S.P.L. (1939). The Proteins in Whey.

[62] Groves M.L. (1960). The Isolation of a Red Protein from Milk2. J. Am. Chem. Soc..

[63] Johanson B. (1960). Isolation of an Iron-Containing Red Protein from Human Milk. Acta Chem. Scand..

[64] Montreuil J., Tonnelat J., Mullet S. (1960). Preparation and properties of lactosiderophilin (lactotransferrin) of human milk. Biochim. Biophys. Acta.

[65] Sanchez L., Calvo M., Brock J.H. (1992). Biological role of lactoferrin. Arch. Dis. Child..

[66] Van der Strate B., Beljaars L., Molema G., Harmsen M., Meijer D. (2001). Antiviral activities of lactoferrin. Antivir. Res..

[67] Levay P.F., Viljoen M. (1995). Lactoferrin: A general review. Haematologica.

[68] Cutone A., Rosa L., Ianiro G., Lepanto M.S., Di Patti M.C.B., Valenti P., Musci G. (2020). Lactoferrin’s Anti-Cancer Properties: Safety, Selectivity, and Wide Range of Action. Biomolecules.

[69] Di Patti M.C.B., Cutone A., Polticelli F., Rosa L., Lepanto M.S., Valenti P., Musci G. (2018). The ferroportin-ceruloplasmin system and the mammalian iron homeostasis machine: Regulatory pathways and the role of lactoferrin. BioMetals.

[70] Rosa L., Cutone A., Lepanto M.S., Paesano R., Valenti P. (2017). Lactoferrin: A Natural Glycoprotein Involved in Iron and Inflammatory Homeostasis. Int. J. Mol. Sci..

[71] Czosnykowska-Łukacka M., Orczyk-Pawiłowicz M., Broers B., Krolak-Olejnik B. (2019). Lactoferrin in Human Milk of Prolonged Lactation. Nutrients.

[72] Rodríguez-Franco D.A., Vázquez-Moreno L., Ramos-Clamont Montfort G. (2005). Actividad antimicrobiana de la lactoferrina: Mecanismos y aplicaciones clínicas potenciales. Rev. Latinoam. Microbiol..

[73] Cheng J.B., Wang J.Q., Bu D.P., Liu G.L., Zhang C.G., Wei H.Y., Zhou L.Y., Wang J.Z. (2008). Factors Affecting the Lactoferrin Concentration in Bovine Milk. J. Dairy Sci..

[74] Sánchez L., Aranda P., Pérez M., Calvo M. (1988). Concentration of Lactoferrin and Transferrin throughout Lactation in Cow’s Colostrum and Milk. Biol. Chem. Hoppe-Seyler.

[75] Yamauchi K., Wakabayashi H., Shin K., Takase M. (2006). Bovine lactoferrin: Benefits and mechanism of action against infectionsThis paper is one of a selection of papers published in this Special Issue, entitled 7th International Conference on Lactoferrin: Structure, Functions, and Applications, and has undergone the Journal’s usual peer review process. Biochem. Cell Biol..

[76] Kell D.B., Heyden E.L., Pretorius E. (2020). The Biology of Lactoferrin, an Iron-Binding Protein That Can Help Defend Against Viruses and Bacteria. Front. Immunol..

[77] Arias M., Hilchie A.L., Haney E.F., Bolscher J.G.M., Hyndman M.E., Hancock R.E.W., Vogel H.J. (2017). Anticancer activities of bovine and human lactoferricin-derived peptides. Biochem. Cell Biol..

[78] Drago-Serrano M.E., Campos-Rodriguez R., Carrero J.C., De La Garza M. (2018). Lactoferrin and Peptide-derivatives: Antimicrobial Agents with Potential Use in Nonspecific Immunity Modulation. Curr. Pharm. Des..

[79] Britigan B.E., Lewis T.S., Waldschmidt M., McCormick M.L., Krieg A.M. (2001). Lactoferrin Binds CpG-Containing Oligonucleotides and Inhibits Their Immunostimulatory Effects on Human B Cells. J. Immunol..

[80] Gibbons J.A., Kanwar R.K., Kanwar J.R. (2011). Lactoferrin and cancer in different cancer models. Front. Biosci..

[81] Legrand D. (2012). Lactoferrin, a key molecule in immune and inflammatory processes1This article is part of Special Issue entitled Lactoferrin and has undergone the Journal’s usual peer review process. Biochem. Cell Biol..

[82] Cohen M.S., Mao J., Rasmussen G.T., Serody J., Britigan B.E. (1992). Interaction of Lactoferrin and Lipopolysaccharide (LPS): Effects on the Antioxidant Property of Lactoferrin and the Ability of LPS to Prime Human Neutrophils for Enhanced Superoxide Formation. J. Infect. Dis..

[83] Chen K., Zhang G., Chen H., Cao Y., Dong X., Li H., Liu C. (2020). Dose Effect of Bovine Lactoferrin Fortification on Iron Metabolism of Anemic Infants. J. Nutr. Sci. Vitaminol..

[84] Ellison R.T., Giehl T.J. (1991). Killing of gram-negative bacteria by lactoferrin and lysozyme. J. Clin. Investig..

[85] Kawakami H., Lonnerdal B. (1991). Isolation and function of a receptor for human lactoferrin in human fetal intestinal brush-border membranes. Am. J. Physiol. Gastrointest. Liver Physiol..

[86] Lönnerdal B., Jiang R., Du X. (2011). Bovine Lactoferrin Can Be Taken Up by the Human Intestinal Lactoferrin Receptor and Exert Bioactivities. J. Pediatr. Gastroenterol. Nutr..

[87] Spik G., Brunet B., Mazurier-Dehaine C., Fontaine G., Montreuil J. (1982). Characterization and properties of the human and bovine lactotransferrins extracted from the faeces of newborn infants. Acta Paediatr..

[88] Liao Y., Jiang R., Lönnerdal B. (2012). Biochemical and molecular impacts of lactoferrin on small intestinal growth and development during early life11This article is part of a Special Issue entitled Lactoferrin and has undergone the Journal’s usual peer review process. Biochem. Cell Biol..

[89] Lepanto M.S., Rosa L., Cutone A., Conte M.P., Paesano R., Valenti P. (2018). Efficacy of Lactoferrin Oral Administration in the Treatment of Anemia and Anemia of Inflammation in Pregnant and Non-pregnant Women: An Interventional Study. Front. Immunol..

[90] Cutone A., Lepanto M.S., Rosa L., Scotti M.J., Rossi A., Ranucci S., De Fino I., Bragonzi A., Valenti P., Musci G. (2019). Aerosolized Bovine Lactoferrin Counteracts Infection, Inflammation and Iron Dysbalance in A Cystic Fibrosis Mouse Model of Pseudomonas aeruginosa Chronic Lung Infection. Int. J. Mol. Sci..

[91] Valenti P., Frioni A., Rossi A., Ranucci S., De Fino I., Cutone A., Rosa L., Bragonzi A., Berlutti F. (2017). Aerosolized bovine lactoferrin reduces neutrophils and pro-inflammatory cytokines in mouse models of Pseudomonas aeruginosa lung infections. Biochem. Cell Biol..

[92] Bezault J., Bhimani R., Wiprovnick J., Furmanski P. (1994). Human lactoferrin inhibits growth of solid tumors and development of experimental metastases in mice. Cancer Res..

[93] Shau H., Kim A., Golub S.H. (1992). Modulation of natural killer and lymphokine-activated killer cell cytotoxicity by lactoferrin. J. Leukoc. Biol..

[94] Iigo M., Shimamura M., Matsuda E., Fujita K.-I., Nomoto H., Satoh J., Kojima S., Alexander D.B., Moore M.A., Tsuda H. (2004). Orally administered bovine lactoferrin induces caspase-1 and interleukin-18 in the mouse intestinal mucosa: A possible explanation for inhibition of carcinogenesis and metastasis. Cytokine.

[95] Chaix J., Tessmer M.S., Hoebe K., Fuséri N., Ryffel B., Dalod M., Alexopoulou L., Beutler B., Brossay L., Vivier E. (2008). Cutting Edge: Priming of NK Cells by IL-18. J. Immunol..

[96] Nakanishi K. (2018). Unique Action of Interleukin-18 on T Cells and Other Immune Cells. Front. Immunol..

[97] Akita K., Ohtsuki T., Nukada Y., Tanimoto T., Namba M., Okura T., Takakura-Yamamoto R., Torigoe K., Gu Y., Su M.S.-S. (1997). Involvement of Caspase-1 and Caspase-3 in the Production and Processing of Mature Human Interleukin 18 in Monocytic THP.1 Cells. J. Biol. Chem..

[98] Kuhara T., Yamauchi K., Tamura Y., Okamura H. (2006). Oral Administration of Lactoferrin Increases NK Cell Activity in Mice via Increased Production of IL-18 and Type I IFN in the Small Intestine. J. Interf. Cytokine Res..

[99] Lorget F., Clough J., Oliveira M., Daury M.-C., Sabokbar A., Offord E. (2002). Lactoferrin reduces in vitro osteoclast differentiation and resorbing activity. Biochem. Biophys. Res. Commun..

[100] Damiens E., El Yazidi I., Mazurier J., Duthille I., Spik G., Boilly-Marer Y. (1999). Lactoferrin inhibits G1 cyclin-dependent kinases during growth arrest of human breast carcinoma cells. J. Cell. Biochem..

[101] Deng M., Zhang W., Tang H., Ye Q., Liao Q., Zhou Y., Wu M., Xiong W., Zheng Y., Guo X. (2012). Lactotransferrin acts as a tumor suppressor in nasopharyngeal carcinoma by repressing AKT through multiple mechanisms. Oncogene.

[102] Hegazy R.R., Mansour D.F., Salama A.A., Abdel-Rahman R.F., Hassan A.M. (2019). Regulation of PKB/Akt-pathway in the chemopreventive effect of lactoferrin against diethylnitrosamine-induced hepatocarcinogenesis in rats. Pharmacol. Rep..

[103] Hayes T.G., Falchook G.F., Varadhachary G.R., Smith D.P., Davis L.D., Dhingra H.M., Hayes B.P., Varadhachary A. (2005). Phase I trial of oral talactoferrin alfa in refractory solid tumors. Investig. New Drugs.

[104] Kuwata H., Yip T.-T., Tomita M., Hutchens T. (1998). Direct evidence of the generation in human stomach of an antimicrobial peptide domain (lactoferricin) from ingested lactoferrin. Biochim. Biophys. Acta (BBA) Protein Struct. Mol. Enzym..

[105] Gifford J.L., Hunter H.N., Vogel H.J. (2005). Lactoferricin. Cell. Mol. Life Sci..

[106] Connor J., Bucana C., Fidler I.J., Schroit A.J. (1989). Differentiation-dependent expression of phosphatidylserine in mammalian plasma membranes: Quantitative assessment of outer-leaflet lipid by prothrombinase complex formation. Proc. Natl. Acad. Sci. USA.

[107] Qasba P.K., Kumar S., Brew K. (1997). Molecular Divergence of Lysozymes and α-Lactalbumin. Crit. Rev. Biochem. Mol. Biol..

[108] Pettersson J., Mossberg A.-K., Svanborg C. (2006). α-Lactalbumin species variation, HAMLET formation, and tumor cell death. Biochem. Biophys. Res. Commun..

[109] Hakansson A., Zhivotovsky B., Orrenius S., Sabharwal H., Svanborg C. (1995). Apoptosis induced by a human milk protein. Proc. Natl. Acad. Sci. USA.

[110] Svensson M., Hakansson A., Mossberg A.-K., Linse S., Svanborg C. (2000). Conversion of alpha -lactalbumin to a protein inducing apoptosis. Proc. Natl. Acad. Sci. USA.

[111] Brinkmann C.R., Heegaard C.W., Petersen T.E., Jensenius J.C., Thiel S. (2011). The toxicity of bovine α-lactalbumin made lethal to tumor cells is highly dependent on oleic acid and induces killing in cancer cell lines and noncancer-derived primary cells. FEBS J..

[112] Sakurai K., Oobatake M., Goto Y. (2008). Salt-dependent monomer-dimer equilibrium of bovine β-lactoglobulin at pH 3. Protein Sci..

[113] Jameson G.B., Adams J.J., Creamer L.K. (2002). Flexibility, functionality and hydrophobicity of bovine β-lactoglobulin. Int. Dairy J..

[114] Ragona L., Fogolari F., Catalano M., Ugolini R., Zetta L., Molinari H. (2003). EF Loop Conformational Change Triggers Ligand Binding in β-Lactoglobulins. J. Biol. Chem..

[115] Reddy I.M., Kella N.K.D., Kinsella J.E. (1988). Structural and conformational basis of the resistance of.beta.-lactoglobulin to peptic and chymotryptic digestion. J. Agric. Food Chem..

[116] Bijari N., Ghobadi S., Derakhshandeh K. (2019). β-lactoglobulin-irinotecan inclusion complex as a new targeted nanocarrier for colorectal cancer cells. Res. Pharm. Sci..

[117] Lišková K., Auty M.A.E., Chaurin V., Min S., Mok K.H., O’Brien N., Kelly A.L., Brodkorb A. (2011). Cytotoxic complexes of sodium oleate with β-lactoglobulin. Eur. J. Lipid Sci. Technol..

[118] Mather I.H., Keenan T.W. (1998). Origin and Secretion of Milk Lipids. J. Mammary Gland. Biol. Neoplasia.

[119] Fong B.Y., Norris C.S., MacGibbon A.K. (2007). Protein and lipid composition of bovine milk-fat-globule membrane. Int. Dairy J..

[120] Kvistgaard A., Pallesen L., Arias C., Lopez S., Petersen T., Heegaard C., Rasmussen J.T. (2004). Inhibitory Effects of Human and Bovine Milk Constituents on Rotavirus Infections. J. Dairy Sci..

[121] Reinhardt T., Lippolis J. (2006). Bovine Milk Fat Globule Membrane Proteome. J. Dairy Res..

[122] Zanabria R., Griffiths M.W., Corredig M. (2020). Does structure affect biological function? Modifications to the protein and phospholipids fraction of the milk fat globule membrane after extraction affect the antiproliferative activity of colon cancer cells. J. Food Biochem..

[123] Clare D.A., Zheng Z., Hassan H.M., Swaisgood H.E., Catignani G.L. (2008). Antimicrobial Properties of Milkfat Globule Membrane Fractions. J. Food Prot..

[124] Aune D., Rosenblatt D.A.N., Chan D.S.M., Vieira A.R., Vieira R., Greenwood D.C., Vatten L.J., Norat T. (2014). Dairy products, calcium, and prostate cancer risk: A systematic review and meta-analysis of cohort studies. Am. J. Clin. Nutr..

[125] Harrison S., Lennon R., Holly J., Higgins J.P.T., Gardner M., Perks C., Gaunt T., Tan V., Borwick C., Emmet P. (2017). Does milk intake promote prostate cancer initiation or progression via effects on insulin-like growth factors (IGFs)? A systematic review and meta-analysis. Cancer Causes Control..

[126] Song Y., Chavarro J.E., Cao Y., Qiu W., Mucci L., Sesso H.D., Stampfer M.J., Giovannucci E., Pollak M., Liu S. (2012). Whole milk intake is associated with prostate cancer-specific mortality among U.S. male physicians. J. Nutr..

[127] Park S.-Y., Murphy S.P., Wilkens L.R., Stram D.O., Henderson B.E., Kolonel L.N. (2007). Calcium, Vitamin D, and Dairy Product Intake and Prostate Cancer Risk: The Multiethnic Cohort Study. Am. J. Epidemiol..

[128] Tate P.L., Bibb R., Larcom L.L. (2011). Milk Stimulates Growth of Prostate Cancer Cells in Culture. Nutr. Cancer.

[129] Qin L.-Q., Wang P.-Y., Xu J.-Y., Li J., Wang J., Sato A. (2006). The Effects of Commercial whole Milk on the Prostate Carcinogenesis in Rats with or without Induction by 2-Amino-1-methyl-6-phenylimidazo[4,5-b]pyridine. J. Heal. Sci..

[130] Fraser G., Jaceldo-Siegl K., Orlich M., Mashchak A., Sirirat R., Knutsen S. (2020). Dairy, soy, and risk of breast cancer: Those confounded milks. Int. J. Epidemiol..

[131] Galván-Salazar H.R., Arreola-Cruz A., Madrigal-Pérez D., Soriano-Hernández A.D., Guzman-Esquivel J., Montes-Galindo D.A., López-Flores R.A., Gomez F.E.-, Rodríguez-Sanchez I.P., Newton-Sanchez O.A. (2015). Association of Milk and Meat Consumption with the Development of Breast Cancer in a Western Mexican Population. Breast Care.

[132] Pala V., Krogh V., Berrino F., Sieri S., Grioni S., Tjonneland A., Olsen A., Jakobsen M.U., Overvad K., Clavel-Chapelon F. (2009). Meat, eggs, dairy products, and risk of breast cancer in the European Prospective Investigation into Cancer and Nutrition (EPIC) cohort. Am. J. Clin. Nutr..

[133] Ji J., Sundquist K. (2015). Lactose intolerance and risk of lung, breast and ovarian cancers: Aetiological clues from a population-based study in Sweden. Br. J. Cancer.

[134] Kong F., Singh R.P. (2010). A Human Gastric Simulator (HGS) to Study Food Digestion in Human Stomach. J. Food Sci..

[135] Schwendel B.H., Wester T.J., Morel P.C., Fong B., Tavendale M.H., Deadman C., Shadbolt N.M., Otter D.E. (2017). Pasture feeding conventional cows removes differences between organic and conventionally produced milk. Food Chem..

[136] Tunick M.H., Van Hekken D.L., Paul M., Ingham E.R., Karreman H.J. (2016). Case study: Comparison of milk composition from adjacent organic and conventional farms in the USA. Int. J. Dairy Technol..

[137] Jahreis G., Fritsche J., Steinhart H. (1997). Conjugated linoleic acid in milk fat: High variation depending on production system. Nutr. Res..

[138] Kuczyńska B., Puppel K., Gołȩbiewski M., Metera E., Sakowski T., Słoniewski K., Gołębiewski M. (2012). Differences in whey protein content between cow’s milk collected in late pasture and early indoor feeding season from conventional and organic farms in Poland. J. Sci. Food Agric..

[139] Tsakali E., Chatzilazarou A., Houhoula D., Koulouris S., Tsaknis J., Van Impe J. (2019). A rapid HPLC method for the determination of lactoferrin in milk of various species. J. Dairy Res..

[140] Fardet A., Rock E. (2018). In vitro and in vivo antioxidant potential of milks, yoghurts, fermented milks and cheeses: A narrative review of evidence. Nutr. Res. Rev..

[141] Paul M., Somkuti G.A. (2010). Hydrolytic breakdown of lactoferricin by lactic acid bacteria. J. Ind. Microbiol. Biotechnol..

[142] Zhang J., Lai S., Cai Z., Chen Q., Huang B., Ren Y. (2014). Determination of bovine lactoferrin in dairy products by ultra-high performance liquid chromatography–tandem mass spectrometry based on tryptic signature peptides employing an isotope-labeled winged peptide as internal standard. Anal. Chim. Acta.

[143] Campanella L., Martini E., Tomassetti M. (2008). New immunosensor for Lactoferrin determination in human milk and several pharmaceutical dairy milk products recommended for the unweaned diet. J. Pharm. Biomed. Anal..

